# Multiple ionization state of Ar^q+^ ions during collisions near the Bohr velocity

**DOI:** 10.1038/s41598-019-41709-w

**Published:** 2019-03-29

**Authors:** Xianming Zhou, Rui Cheng, Yongtao Zhao, Yuyu Wang, Yu Lei, Yanhong Chen, Xinwen Ma, Guoqing Xiao

**Affiliations:** 1 Institute of Modern Physics, Chinese Academy of Sciences, Lanzhou, 730000 China; 2 School of Science, Xi’an Jiaotong University, Xi’an, 710049 China

## Abstract

In order to clarify the mechanism and the influence of the initial charge state and target atomic parameters for the formation of L-shell multiple ionization state of Ar^q+^ ions produced by the collisions near the Bohr velocity, the k-shell x-ray emission of Ar is measured for 1.2 MeV Ar^q+(q=4, 6, 8, 9, 11, 12)^ ions impacting on V target and 3 MeV Ar^11+^ ions interacting with selected targets (Z_2_ = 23, 26, 27, 28, 29, 30). It is found that the measured Ar Kα and Kβ x-ray energies shift to the high energy side, and the relative intensity ratios of Kβ/Kα are enlarged than the atomic data, owing to the presence of out-shell multiple vacancies. The multiple ionization is almost independence of the projectile charge state, but is diminished with increasing target atomic number.

## Introduction

The interaction of highly charged ions (HCI) with solid target has been intensively investigated in recent years, not only for the significance of basic researches such as, astrophysics, atomic physics and ion-atom collisions reactive dynamics under extra conditions, but also for the requirement of practical applications in many fields, i.e., biomedicine, material science and new energy development technology^1–5^. X-ray emission, which is one of the important consequential results from ion-atom collisions, contains important information of several inner-shell processes as primary ionization, vacancy decay and intra-atomic excitation, and provides a valid method to investigate the particle properties and complex interaction mechanism^6,7^. For example, many works have been carried out focusing on the excitation and decay of the highly charged Ar ions by analyzing the K-shell x-ray emission^8–21^.

In the lower energy region, the kinetic energy of HCI is too small to induce the direct ionization of inner-shell electrons, and the resulting interaction is mainly the neutralization of projectile in the vicinity of the surface by the charge exchange between the ion and target atom^22^, and that can be well described by the classical over-barrier model (COBM)^23^. In the high energy region, because the time, in which the swift heavy ion passes through the target region which is equal to the attenuation length of the characteristic x ray, is generally less than that of the de-excitation of its vacancies, the decay of the incident ions cannot be observed. The radiation measurement offers mainly the information of the target atom ionization. Lots of works have been done to understand such behavior in experiment^24–26^, and many theories, such as, binary encounter approximation (BEA)^27^, plane wave Born approximation (PWBA)^28^ and energy-loss Coulomb-repulsion perturbed-stationary-state relativistic (ECPSSR)^29^, have been well established to simulate this process. However, duo to the complicated collisions process and the limitation of experimental conditions, the investigation of evolution of the projectile during the interaction near the Bohr velocity is nearly absent.

In fact, in the energy region near the Bohr velocity, the projectile not only has enough time to capture electrons from the target atom to neutralize, but also has enough kinetic energy to bombard the target atom at a close distance below the surface to ionize the orbital electrons. In addition to the inner-shell single ionization, under the dual effects of neutralization and ionization, the projectile may remain multiple-ionization state in the outer shells when the inner-shell x-ray emission occurs. This results in the blue shift of the corresponding x-ray energy and the change of the relative intensity ratio of the sub-shell x ray. In our previous work, such multiple ionization of projectile has been observed and the effect of incident energy was verified^30^. Here, we would like to present the further research, and the special attention will be devoted to the influence of projectile charge state and target atomic number on the multiple ionization.

## Experimental Method

**Figure 1:**
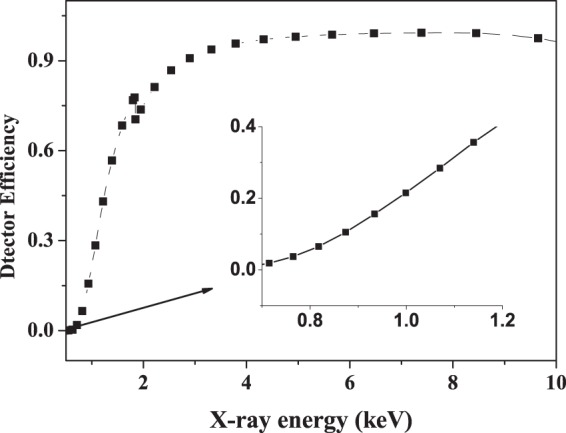
Efficiency curve of the Silicon Drift Detector. The curve is a fifth polynomial in the 0.6–1.8 keV energy interval, a fourth polynomial in the 1.8–4.0 keV energy interval and a third polynomial in the 4.0–10 keV energy interval.

The measurements have been carried out at the 320 kV high voltage experimental platform at Institute of Modern Physics, Chinese Academy of Sciences (IMP, CAS) in Lanzhou. More details of the experimental system have been described in the previous work^31^. In brief, the argon ions are produced and extracted from the Electron Cyclotron Resonance (ECR) ion source and selected by a 90° analyzing magnet, and then introduced into the ultrahigh vacuum target chamber (10^−8^ mbar) after acceleration, focus, multi-deflections and multi-collimations. The divergence of the beam is smaller than 0.2°. The ion beam impacts perpendicularly onto the target with spot size of about Φ3 mm. The target, having a purity of 99.99% with surface area of 15 × 20 mm^2^ and thickness of 0.1 mm, is positioned on a sample holder. It permits a three-dimensional movement to change the target position freely and remove away from the beam line in order to measure the current. The x-rays are detected by a silicon drift detector (SDD) which has an effective detection area of 7 mm^2^ and a 12.5 μm Beryllium window in the front of the detector. The SDD is placed at 80 mm far away from the target surface in the chamber and at 135° to the beam direction. The detector has an effective energy range of 0.5–14.3 keV when the gain was selected at 100, and an energy resolution of about 136 eV at 5.9 keV. The energy calibration is done using simultaneously the two standard radioactive sources of ^55^Fe and ^241^Am, and then tested by measuring the K-shell x rays of Al, V and Fe produced by proton impact. In this way, that can guarantee a precise measurement of the x-ray energy. The SDD intrinsic efficiency, which combines the effects of transmission through the Beryllium window and interaction in the silicon detector, was well determined by transmission measurement. As shown in Fig. 1, the efficiency curve can be described with different polynomial in various energy intervals. The number of incident projectiles, which could not be measured immediately by recording the target current due to the influence of the secondary emission, is detected indirectly by the combined use of a penetrable Faraday cup and a common one.

## Results and Discussion

### K x-ray emission of Ar^q+^ ions

**Figure 2:**
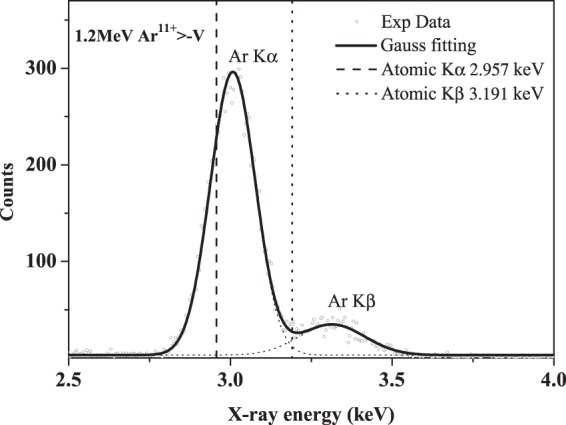
Typical argon K-shell x-ray spectra for Ar^11+^ ions impacting on vanadium targets.

Figure 2 presents a typical x-ray spectra of argon produced from the interactions of Ar^q+^ ions with the selected solid targets in the energy region near the Bohr velocity, and which are well fitted by a Non-linear Curve Gaussian fitting program, taking into account the detector efficiency. Two distinct peaks are obtained and identified as the Kα and Kβ x-ray of Ar, which originate from the decay of K-shell vacancy with 2p and 3p electrons, respectively. Because the flying time of the projectile from the ion source to the target surface, which is about 3.4 × 10^−6^ s for 3 MeV Ar, is long enough for the decay of all the original metastable state, the present results can be eliminated for the decay of metastable state above the surface^30^. For the all Ar^q+^ ions in the present work, there is no initial k-shell vacancy. However, this is prerequisite for the K x ray emission. Therefore, the appearance of the K x-ray indicates that the Ar K-shell electron is ionized during the interaction of Ar^q+^ ion with target atom^32^. According to the previous investigation^16^, the K shell is mainly ionized by direct coulomb collision, and that can be well simulated by BEA the corrections of coulomb repulsion and ionic binding energy. In addition, except for Ar^q+^ ions having two M_2_ electrons, there is no 3 P electrons for other ions as Ar^q+ (q=6, 8, 9, 11, 12)^, which is directly related to the Kβ radiation. The emission of Kβ shows that the projectile undergoes a neutralization process by electron capture. In summary, the experimental x-ray is the dual result of ionization and capture, and is emitted after the collisions below the surface.

One can find in Fig. 2 that the experimental value of the K x-ray energy is larger than that of the single ionized atom. For example, the blue shift of Kα x-ray is about 51 ± 3 eV for 1.2 MeV Ar^11+^ ions impacting on vanadium, and that of Kβ is about 123 ± 5 eV. As mentioned above, although the impact velocity of Ar ions is large, the x-ray emissions occur mainly after the collisions below the surface, where the projectile has been slow down. Therefore, the influence of Doppler shift on the blue shift can be neglected.

So, it is proposed that this energy shift can be attributed to the effect of the multiple ionization state of the L shell^30,33^. Because, in this case, owing to the absence of the outer-shell electrons, the screening of the nuclear is weakened, and the binding energy of the remaining electrons is perturbed. As a result, the observed x-ray energy shifts to the high energy side. In the present work, the Ar^q+^ ions undergo simultaneously the dual effects of ionization and electron capture during the interaction with targets atom. In addition to the neutralization, the Ar^q+^ ions are also ionized. Not only the k-shell is ionized, but also the L-shells are multiply ionized. At the balance of ionization and electron capture, the L-shell remains multiple ionization state when the K-shell x-ray emission occurs. This leads to the blue shift of the K x-ray energy.

### Charge state effect on the multiple ionization state of Ar^q+^ ions

On the one hand, the initial charge state of incident ions determines the number of remaining electrons and their binding energy, and which in turn affects the corresponding ionization cross section. On the other hand, it determines its own potential energy and affects the probability of electron capture. Generally, the higher the charge state of projectile, the larger the binding energy of the residual orbital electrons is, and the smaller the ionization probability is, however, the higher the cross section of electron capture is. During the collisions near the Bohr velocity, with the influence of ionization and neutralization, the multiple ionization state of the projectile will be affected by the initial charge state.

**Figure 3:**
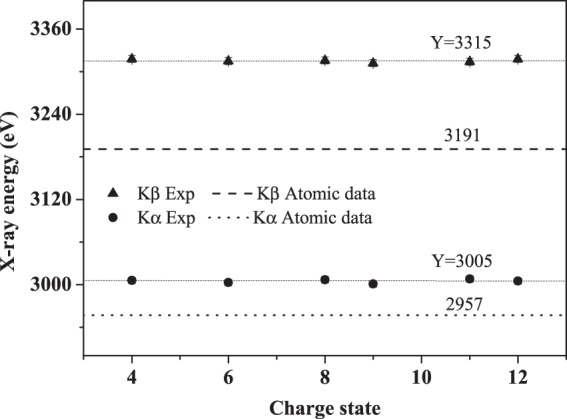
Measured energies of Ar Kα and Kβ x-ray as a function of the charge state.

Figure 3 presents the experimental energies of Ar Kα and Kβ x-ray. With the increase of the initial charge state, this did not change substantially within the range of experimental error. The average value of Kα x-ray is about 3005 ± 3 eV, and that of Kβ is about 3315 ± 5 eV. Compared to the atomic date, the two lines shift to the high energy side of about 56 ± 3 eV and 126 ± 5 eV, respectively. Such results allow us to estimate that there are about three 2p vacancies when the K x-ray emissions occur, which is similar as the results of Ar^11+^ ions^33,34^. This indicates that regardless of the projectile initial charge state, namely, regardless of the initial electron configuration of the L and M shells, during the collisions near the Bohr velocity, the arrangement of projectile L shell will reach a same station under the two actions of electron ionization and capture. The multiple ionization state of Ar L shell is independent of the initial charge state.

Another thing can be deduced from Fig. 3 is that the three vacancies on 2p shell for Ar^11+^ ions after collision is not the retention of the initial charge state, but is the synergistic result of ionization and capture. For example, for Ar^11+^, such two actions happen to be in dynamic equilibrium, as a result, the number of 2p electron seems to have no change. For Ar^12+^ ions, whose 2 P shell is in a state of less than half-full, the charge exchange is stronger than the ionization, and this leads to the increase of L electrons. For Ar^4+~9+^ ions, whose 2p shell has more 3 electrons, the probability of ionization is higher than that of capture, and this results in the decrease of L electrons.

**Figure 4:**
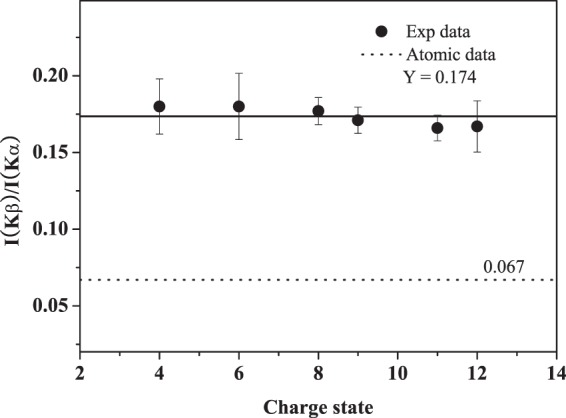
Intensity ratios of Ar Kβ to Kα x-ray as a function of the charge state.

The relative intensity ratios of Ar Kβ to Kα X-ray are given in Fig. 4 as a function of the initial charge state, which are all larger than the atomic data. This result provides another evidence for the L shell multiple ionization state of Ar ions. Because, for the decay of K shell vacancy, the sum of the fluorescence yield ω and radiationless yield *a* is unity^35^, ω_Κ_ (ω_Κ_α + ω_Κ_β) + *a*_K_ = 1. Under the L-shell multiple ionization, the probability of KLL Auger transition is decreased owing to the absence of L electrons, and the K-L radiation transition, namely Kα X-ray emission, is reduced because this x-ray is directly related to 2p electrons. Therefore, K-M radiation transition, Kβ X-ray emission, is enhanced. As a result, the actual intensity ratio of Kβ to Kα X-ray is enlarged.

### Target atomic number on the multiple ionization state of Ar^11+^ ions

When the same incident ion bombard on the different targets, for the ionization of the orbital electrons of projectile, the relative effective collision energy is diverse, and the ionization cross sections are also distinct. The binding energy of various atoms is diverse, and this leads to the diversity of the electron capture probability for projectile neutralization. So, target parameter will have a certain impact on the formation of multiple ionization state of the projectile.

**Figure 5:**
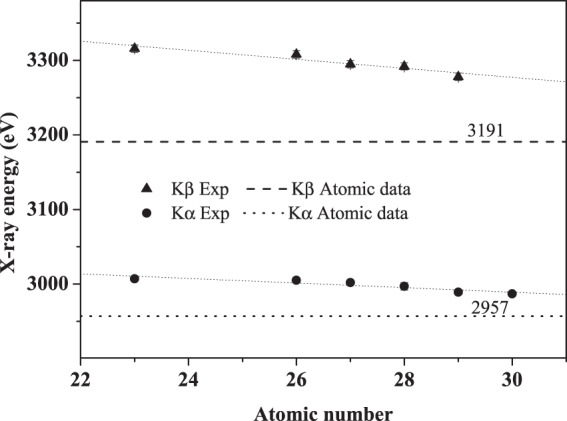
Measured energies of Ar Kα and Kβ x-ray as a function of the target atomic number.

Figure 5 shows the Ar K x-ray energies for 3 MeV Ar^11+^ ions impacting on various targets. The experimental results of Kα and Kβ are all higher than the atomic data, and they decrease with the increase of the target atomic number Z_2_. For Z_2_ from 22 to 30, Kα is diminished from 3.007 to 2.987 keV, and that is for Kβ from 3.316 to 3.278 keV, respectively. The result indicates that, during the collision with various targets, the Ar ions generate the K-shell ionization and forms the L and M shell multiple ionization state, and the extent of such multiple ionization is reduced as a function of the target atomic number.

**Figure 6:**
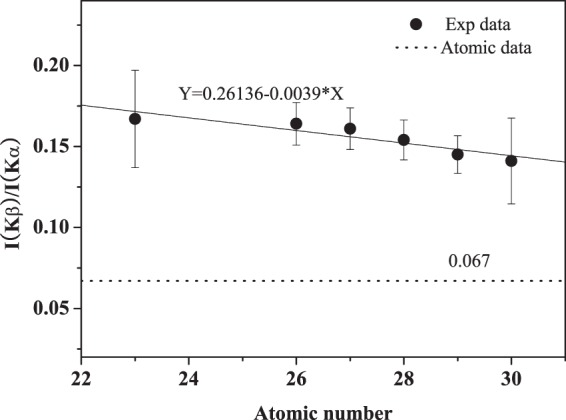
Intensity ratios of Ar Kβ to Kα x-ray as a function of the target atomic number.

In order to further clarify the above deduction, the relative intensity ratios of Kβ to Kα x-ray are also investigated. As shown in Fig. 6, the ratios are all larger than the atomic data of 0.067 on various targets, and this is dwindled as the target atomic number increases. This is similar to the conclusion in Fig. 4, namely, the extent of the multiple ionization state is decreased with increasing target atomic number.

This result can be easily understood from the point of neutralization process of projectile. The average binding energy of M shell electrons for Ar^11+^ ions is about 100 eV. For the target from V to Zn, the binding energy of the M shell electrons is about 50~90 eV, and that for the L shell electrons is about 530~1040 eV, respectively. Following the principle of energy level matching, the de-excitation of Ar^11+^ ions is mainly to capture electrons from the M shells of the target atom to fill their own M shell holes, and then to fill the L and K shell vacancies by the transition of M shell electrons. With the increase of target atomic number, the number of M shell electrons increases, and the energy level matching between the projectile and target is enhanced. Therefore, the filling rate of M shell holes for the projectile is increased. Correspondingly, the decay of the L shell holes is enhanced. As a result, this reduces the multiple ionization on L shell, and leads to the decrease of the blue shift and the relative intensity ratio of the observed x-ray.

## Conclusions

The K x-ray emission of argon has been investigated as a function of the initial charge state and target atomic number for Ar^q+^ ions impacting on various targets. The result indicates that, in collision near the Bohr velocity, the projectile undergoes the process of ionization, besides the neutralization by electron capturing. Not only the k-shell ionization, but also the outer-shells are also multiply ionized. Under the synergistic action of ionization and vacancy decay, the L shell is in a multiple ionization state when the K x-ray is emitted. The extent of such multiple ionization is almost invariant with the projectile charge state, but decreases as the target atomic number increases. This causes the blue shift and the enhancement of the intensity ratio of Kβ and Kα x-ray.
